# Activation of a Bacterial Mechanosensitive Channel, MscL, Underlies the Membrane Permeabilization of Dual-Targeting Antibacterial Compounds

**DOI:** 10.3390/antibiotics11070970

**Published:** 2022-07-19

**Authors:** Robin Wray, Junmei Wang, Paul Blount, Irene Iscla

**Affiliations:** 1 Department of Physiology, University of Texas Southwestern Medical Center, 5323 Harry Hines Blvd., Dallas, TX 75390, USA; robin.wray@utsouthwestern.edu; 2 Computational Chemical Genomics Screening Center, Department of Pharmaceutical Sciences, School of Pharmacy, University of Pittsburgh, Pittsburg, PA 15261, USA

**Keywords:** bacterial channels, antibiotic resistance, bacterial drug target, dual mechanism antibiotics, druggable target

## Abstract

Resistance to antibiotics is a serious and worsening threat to human health worldwide, and there is an urgent need to develop new antibiotics that can avert it. One possible solution is the development of compounds that possess multiple modes of action, requiring at least two mutations to acquire resistance. Compound SCH-79797 both avoids resistance and has two mechanisms of action: one inhibiting the folate pathway, and a second described as “membrane permeabilization”; however, the mechanism by which membranes from bacterial cells, but not the host, are disrupted has remained mysterious. The opening of the bacterial mechanosensitive channel of large conductance, MscL, which ordinarily serves the physiological role of osmotic emergency release valves gated by hypoosmotic shock, has been previously demonstrated to affect bacterial membrane permeabilization. MscL allows the rapid permeabilization of both ions and solutes through the opening of the largest known gated pore, which has a diameter of 30 Å. We found that SCH-79797 and IRS-16, a more potent derivative, directly bind to the MscL channel and produce membrane permeabilization as a result of its activation. These findings suggest that possessing or adding an MscL-activating component to an antibiotic compound could help to lower toxicity and evade antibiotic resistance.

## 1. Introduction

Soon after the discovery of antibiotics, the ability of bacteria to mutate and generate resistance to them was reported (see Levy and Marshall [1] for a review). Despite early warnings about the perils of their overuse, widespread overprescription of antibiotics and their use in agriculture resulted in a rise in bacteria resistance to all available therapeutics, often called super-bugs. Therefore, efforts to address the current antibiotic resistance crisis are focused on increasing the stewardship of our current antibiotics and the development of novel treatments. Of special interest are antibiotics with a decreased tendency to produce resistance in bacteria. One promising possibility appears to be antibiotics with multiple mechanisms of action or combination therapies that generate less resistance because two independent mechanisms have to be overcome [2,3].

**Figure 1:**
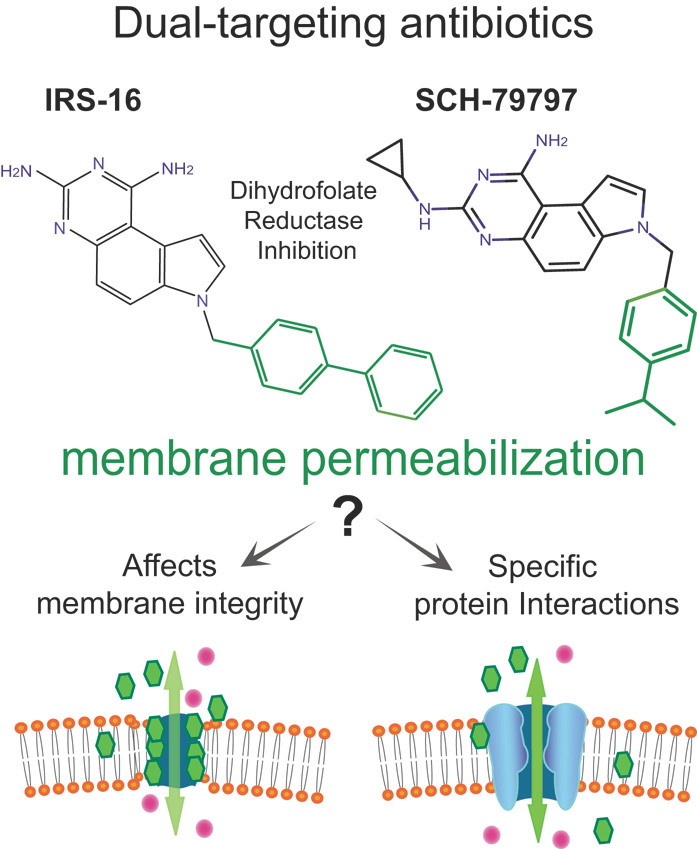
Deciphering the “membrane permeabilization” mechanism of two novel dual-target antibiotics. Structures of the antibacterial compound SCH-79797 and its derivative IRS-16 and their dual-targeting mechanism of action. The mechanisms of action of each component of the compounds have been described: one targeting the folate metabolism (black) by inhibiting the dihydrofolate reductase, and the other increasing membrane permeabilization (green). Here, we elucidate whether this latter effect is non-specific (**bottom left**) or dependent on the expression of bacteria-specific channels (**bottom right**).

One recently described novel antibiotic compound, SCH-79797 [4], not only was effective against antibiotic-resistant strains but also proved very difficult for bacteria to develop resistance to it.A dual-targeting mechanism of action was elucidated for this compound: one is by inhibition of the dihydrofolate reductase (folic acid metabolism inhibition), and the other was simply described as an increased membrane permeability and depolarization [5]. Furthermore, a derivative, Irresistin-16 (IRS-16), had increased potency, less toxicity, and was effective against Neisseria gonorrhoeae in a mouse vaginal infection model. While membrane permeabilization was well described as one of its modes of action, the exact mechanism(s) remained obscure (Figure 1).

Mechanosensitive (MS) channels in bacteria serve the physiological role of osmotic emergency release valves (see [6,7] for recent reviews). When bacteria are exposed to high osmolarity, they transport (K^+^, glutamate, betaine, and proline) and synthesize (glutamate, trehalose, proline, and betaine) solutes in the cytoplasm to accommodate the increase in external osmolarity. Such accommodation keeps the cell turgor high, which is a requisite for cell growth and division [8]. However, when the osmotic environment acutely decreases (e.g., when it rains), water rushes into the cell, the turgor increases, and tension builds in the membrane, which, if not reversed, could damage it, leading to decreased cell viability. However, the membrane tension gates the two primary bacterial MS channels, MscS (mechanosensitive channel of small conductance) and the unrelated MscL (mechanosensitive channel of large conductance), which function redundantly, allowing for a rapid discharge of cytoplasmic solutes and the cell to reach osmotic homeostasis [9]. 

The membrane-tension-gated channel with the largest pore, MscL, is unique to microbes, highly conserved, and found in the vast majority of Gram-negative and Gram-positive species, including most pathogens. Opening the largest gated pore known, MscL allows the passage of molecules up to 30 Å in diameter [10,11], not only letting solutes out but also antibiotics in [6,12,13,14]; not surprisingly, inappropriate gating of the channel is detrimental to the cell [6,15,16]. 

From the original studies, it could be presumed that both SCH-79797 and IRS-16 directly disrupt the membrane, leading to permeabilization (see Figure 1, bottom left). However, since the membrane permeabilization effects are bacterial specific, as suggested by the low toxicity observed in animal models [4,5,17,18], we hypothesize that they could be targeting bacterial-specific channels, thus explaining its microbial specificity (Figure 1, bottom right). Here, we find that direct activation of the bacterial mechanosensitive channel MscL underlies the membrane permeabilization effect of the antibiotic compound SCH-79797 and its derivative IRS-16. We show direct channel activation of MscL by these compounds in patch clamp and define a binding pocket using molecular docking and mutational experiments. Together, our data define a mechanism of action for these compounds and suggest that targeting MscL is a desirable additional mechanism of action for antibacterial compounds to both increase their bacterial specificity and lower rates of acquired resistance.

## 2. Results

### 2.1. SCH-79797 Antibiotic Effects in E. coli Are MscL Dependent

**Figure 2:**
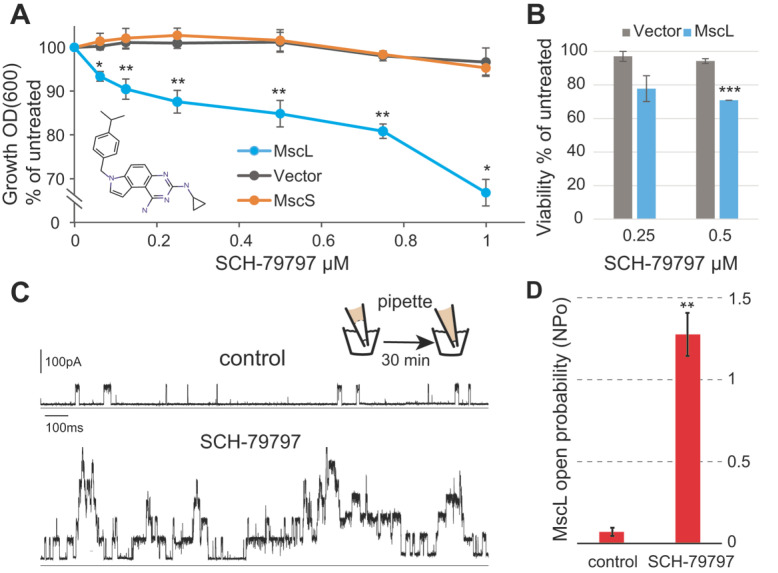
The antibiotic effects of SCH-79797 in vivo are dependent on MscL expression. (**A**) *E. coli* strain MJF455 (ΔMscS, ΔMscL) cultures carrying an empty vector (gray), expressing MscS (orange), or expressing MscL (cyan) were treated with increasing concentrations of the compound SCH-79797 (insert). The graph represents growth inhibition as a percentage of the non-treated cultures (*n* = 4) * *p* < 0.05, ** *p* < 0.005. (**B**) Viability of *E. coli* MJF455 cells, carrying an empty plasmid (gray) or expressing MscL (cyan) after treatment with SCH-79797. Results are graphed as a percentage of the untreated cultures *** *p* < 0.0001 Student *t*-test MscL vs. vector. (**C**) Representative traces of MscL activity recorded by a patch clamp on native bacterial membranes. Experiments were performed using the back-filled pipette configuration (scheme), where channel activity was measured shortly after patch formation (control, **upper trace**) and then again after diffusion of the compound to the pipette tip (**lower trace**). The same patch was recorded at the same negative pressure before and after treatment. (**D**) Quantification of the channel activity is shown as the probability of opening (Npo) of the MscL channel before and after treatment while held at the same pressure (*n* = 4). ** *p* < 0.003 Student *t*-test paired treated vs. untreated.

Since membrane permeabilization by SCH-79797 seems to be specific to bacteria [4,5], we first evaluated whether the SCH-79797 antibiotic effects were dependent on the expression of bacterial MS channels. For this purpose, we used the *E. coli* strain MJF455, null for the two major MS channels, MscS and MscL, carrying an empty vector or expressing in trans either of the channels. Note that MscS, another bacterial mechanosensitive channel from an independent family, also senses membrane tension and is even more sensitive than MscL to compounds that modify membrane properties [6,9,19], and thus serves as a control for MscL specificity. As shown in Figure 2A, inhibition of bacterial growth that correlated with the SCH-79797 concentration was observed only when MscL was expressed. In contrast, cultures of bacteria carrying an empty plasmid or expressing MscS were not affected at concentrations under 1 uM. When the effect of SCH-79797 on viability was measured, similar results were obtained (Figure 2B), where only MscL-expressing cultures were affected by SCH-79797 treatment. Consistent with SCH-79797’s dual-targeting mechanism of action, at higher concentrations, even MscL-null strains were affected (Supplementary Figure S1A); folate pathway inhibition, the other mode of action, was intact in these strains. On the other hand, cumene, the part of the SHC-79797 molecule shown to be responsible for the membrane permeabilization effect [5] (Figure 1), exclusively affected cells expressing MscL, even at concentrations as high as 15 µM. Cells expressing MscS were not affected by cumene nor were cells carrying an empty vector (Figure S1B). These results suggest that some of the effects of SCH-79797 and all of cumene are MscL specific and not simply due to a general membrane disruption.

To demonstrate a more direct influence on MscL, we further examined the effect of SCH-79797 on MscL activity by patch clamp in native bacterial membranes using the giant-cell or spheroplast preparation [20]. The specific technique used back-filling of the pipette (Figure 2C inset), allows the presentation of the compound from the periplasmic side of the cell. The tip of the pipette was filled with drug-free buffer (control), and the rest of the pipette was back-filled with a 5 µM SCH-79797 solution. Because it took some time for the compound to reach the tip of the pipette, this configuration also allowed a comparison of the same patch before and after treatment. In this approach, enough negative pressure was applied to the patch to begin to elicit MscL activity. The same patch was activated at the same pressure after 30 min, after the compound diffused to the tip of the patch. This approach has been found to be reproducible, specific, and reliable for testing the influence of pH shifts and compounds on MscL activity [13,14,20,21,22,23,24,25]. Typical electrophysiology traces of MscL activity are shown in Figure 2C. Upper deflections in the current trace represent MscL openings. Upon diffusion of SCH-79797, channel activity was greatly increased. This phenomenon was observed in all patches backfilled with 5 µM SCH-79797 but not in controls backfilled with the 1% DMSO carrier only. Quantification of the MscL channel activity from traces before and after treatment with SCH-79797 is shown in Figure 2D. The probability of opening (Np_o_) of the MscL channel showed a significant increase in MscL activity after treatment of SCH-79797 (Figure 2C,D). These experiments are in agreement with the observed MscL dependency of the SCH-79797 effects in vivo, and further support that the mechanism of action described as “membrane permeabilization” is indeed due to the activation of MscL. 

### 2.2. IRS-16 Antibiotic Effects in E. coli Are MscL Dependent

**Figure 3:**
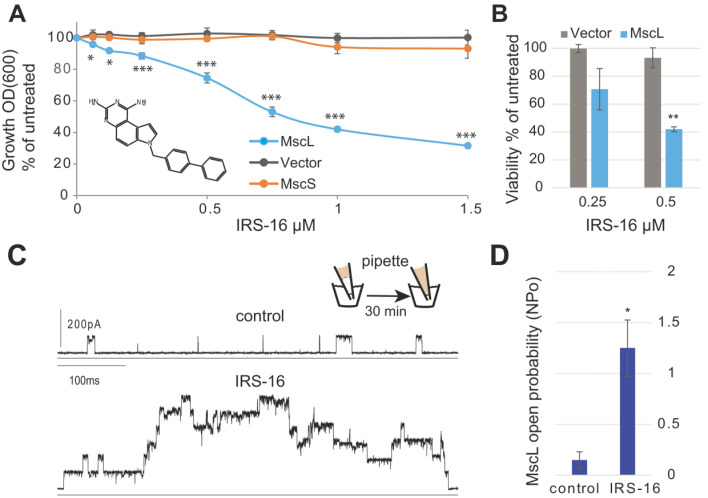
The antibiotic effects of IRS-16 in vivo are dependent on MscL expression. (**A**) *E. coli* strain MJF455 (ΔMscS, ΔMscL) cultures, carrying an empty vector (gray), expressing MscS (orange), or expressing MscL (cyan) were treated with increasing concentrations of the compound IRS-16 (insert). The graph represents growth inhibition as a percentage of the non-treated cultures (*n* = 4) * *p* < 0.02, *** *p* < 0.0005. (**B**) Viability of *E. coli* MJF455 cells, carrying an empty plasmid (gray) or expressing MscL (cyan) after treatment of IRS-16. Results are graphed as a percentage of the untreated cultures ** *p*< 0.003 Student *t*-test MscL vs. vector. (**C**) Representative traces of the MscL activity recorded by a patch clamp on native bacterial membranes. Experiments were performed using the back-filled pipette configuration (scheme), where channel activity was measured shortly after patch formation (control, **upper trace**) and then again after diffusion of the compound to the pipette tip (**lower trace**). The same patch was recorded at the same pressure before and after treatment. (**D**) Quantification of the channel activity is shown as the probability of opening (Npo) of the MscL channel before and after treatment while held at the same pressure (*n* = 4). * *p* < 0.05 *t*-test paired treated vs. control.

Similar experiments were performed using IRS-16, a SCH-79797 derivative with lower toxicity that is effective against gonorrhea infection in a mouse model [4,5]. Again, as can be seen in Figure 3, panels A and B, the IRS-16 in vivo antibiotic effects were clearly MscL dependent, both for growth inhibition and viability. Similar to SCH-79797, in the patch clamp experiments using native bacterial membranes, MscL activity significantly increased after treatment with IRS-16. 

Consistent with previous results [5], IRS-16 proved to be more potent than SCH-79797 against our *E. coli* strain (Figure 2 and Figure 3) and more MscL specific at higher concentrations (Figure S1C). Consistently, we found that IRS-10, the part of the molecule targeting the dihydrofolate reductase that did not cause membrane permeabilization, showed no MscL dependency (Figure S1D). 

### 2.3. Determination of the SCH-79797-Binding Site to MscL 

**Figure 4:**
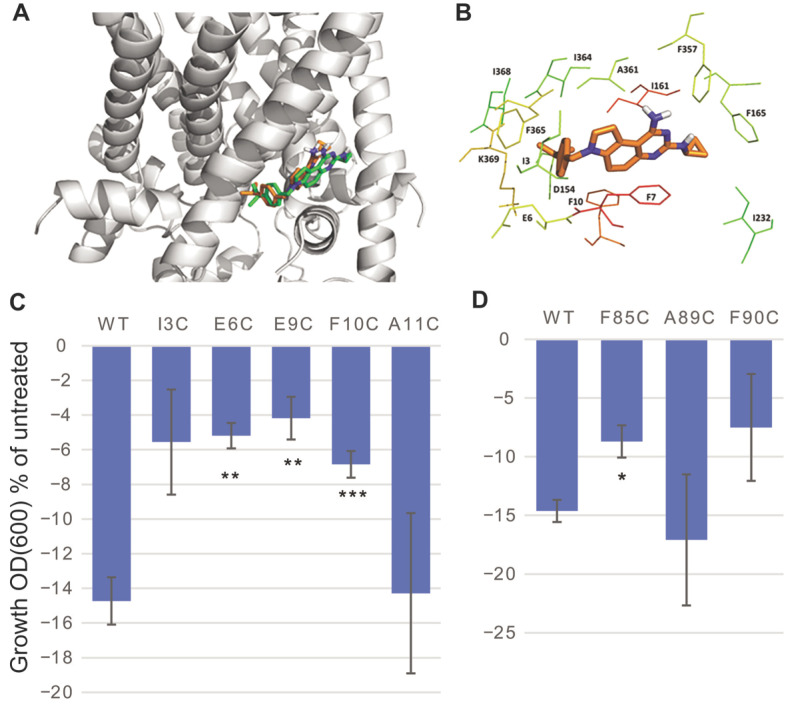
Data indicating a binding pocket for SCH-79797 to MscL. (**A**) Docking (green sticks) and representative MD (brown sticks) binding poses for SCH-79797. (**B**) The key residues ($∆G_{MM-GBSA}^{lig-res}<-0.5$ kcal/mol). The redder a residue is, the stronger the ligand–residue interactions, and the greener a residue is, the weaker the ligand–residue interactions. (**C**) Growth inhibition of MJF455 cultures expressing Eco MscL WT and N-terminal mutants, after treatment with 1µM SCH-79797, expressed as a percentage of the untreated cultures. (**D**) C-terminal mutants. Student test non-paired vs. WT MscL *n* ≥ 3, * *p* ≤ 0.05, ** *p* ≤ 0.005, *** *p* ≤ 0.0005.

In order to determine if there was specific binding, and if so, the binding site of SCH-79797 to the MscL protein, we used methods that have previously proved successful in finding the interactions of other agonist compounds with MscL [12,13,14,25]. The first step was to use the Glide module of the Schrodinger’s software to determine where the ligands would dock within the protein. As the top docking pose may not necessarily reflect the actual binding pose, we studied several top yet distinct docking poses (two for IRS-16 and three for SCH-79797). The most favorable binding pose for a ligand was the one that has the lowest MM-PBSA-WSAS free energy. MD simulations and free energy decomposition experiments were performed for SCH-79797 and IRS-16 and collectively, the data suggested a binding site at the interface of the N-terminal residues (S1) and the cytoplasmic portion of the second transmembrane domain (TM2) of MscL as shown in Figure 4A,B. The time courses of the root-mean-square deviations (RMSDs) are shown in Figure S2 and the free energy terms of the binding and specific residues involved in this binding pocket and their individual contribution to the binding are summarized in Tables S1 and S2, respectively. The binding site we found for the SCH-79797 compound is analogous but not identical to the known binding sites of other compounds. Even the binding patterns of SCH-79797 and IRS-16 show differences: the bi-phenyl group in IRS-16 has more contact with the lipid than its counterpart, the cumene group in SCH-79797, and according to the free energy analysis, IRS-16 can bind tighter than SCH-79797 (Table S1), which might explain why the former is more potent and more bacteria specific.

Mutational analysis of some of the residues within the region was performed to experimentally test the putative binding site of SCH-79797. Some of the residues with better docking scores are known to be crucial for channel gating, leading to strong loss-of-function (LOF) phenotypes when substituted [26,27,28]. Because these LOF mutants are more difficult to gate and the results are difficult to interpret, they were excluded. As shown in Figure 4C,D, cells expressing MscL channels with mutations of several of the residues predicted to be in the region of SCH-79797 binding showed a significantly altered response to treatment when compared to WT MscL-expressing cells. E6 and F10 were also less sensitive to IRS-16 (Figure S3). These data are consistent with these residues being at or near the binding pocket and that SCH-79797 and IRS-16 have overlapping binding sites.

**Figure 5:**
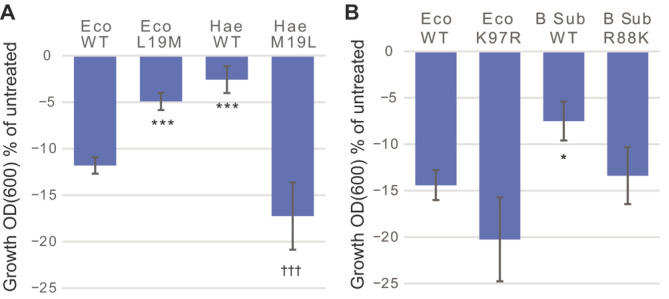
Effect of SCH-79797 on bacteria expressing MscL orthologues and counter mutants. Growth inhibition of MJF455 cultures expressing different constructs after treatment with 1µM SCH-79797, expressed as the percentage of the untreated cultures. (**A**) WT Eco MscL, Eco L19M, Hae WT, or Hae M19L. (**B**) WT Eco MscL, Eco K97R, B Sub WT, or B Sub R88K. *n* ≥ 3 * *p* ≤ 0.03, *** *p* ≤ 0.0002, *t*-test non-paired against WT Eco, ††† *p* ≤ 0.0005, *t*-test non-paired against WT Hae.

A second method used in this study compared the response to SCH-79797 treatment of cells expressing MscL orthologues with naturally occurring differences within the proposed binding pocket. If, as predicted, cells expressing orthologues without the canonical-binding site are less sensitive to the compound, site-specific mutations can be made to generate a better canonical-binding site. If these mutated channels result in increased sensitivity to the compound, the most likely interpretation is that the channel now binds the compound better. Such experiments allow one to further confirm the binding pocket. As shown in Figure 5A, we tried the orthologue *Haemophilus influenzae* (Hae MscL), which has a critical difference within the proposed binding area: L19 in *E. coli* is M19 in *H. influenzae*. Note that even though this residue was not predicted to directly intervene in the binding site, *E. coli* D18, the neighboring residue, was; however, could not be studied in vivo because mutations at this site lead to strong loss-of-function phenotypes. Thus, differences at the L19 site may act directly or allosterically within the canonical-binding pocket. As we predicted, the response to treatment was significantly reduced for cells expressing *H. influenzae* MscL. When the counter mutations were studied, the experiments confirmed the importance of this site in effecting a response to SCH-79797. 

The residue K97 is also predicted to contribute to the SCH-79797-binding pocket. *Bacillus subtilis* (B. sub) has a naturally occurring change at the equivalent location, R88. As seen in Figure 5B, substitution of K with R at the 97 position of *E. coli* did not significantly change the effectiveness of SCH-79797. *B. subtilis* did not respond as well to SCH-79797 as *E. coli*, but the conservative mutation of R to K at position 88 led to a channel that was as sensitive to SCH-79797 as *E. coli*, suggesting again that this residue contributes to SCH-79797 binding.

Collectively, molecular docking, mutagenesis, and study of the *H. influenzae* and *B. subtilis* orthologues and their mutants all suggest that SCH-79797 activates MscL by specifically binding to the channel within a specific site at the interface between subunits at the cytoplasmic–membrane region of the protein. 

## 3. Discussion

One obvious way to avoid antibiotic resistance is to attack the bacteria with a drug, or combination of drugs, that has more than one mode of action [29]. SCH-79797 and IRS-16 are such compounds and, as predicted, bacteria do not easily acquire resistance to them [5]. One of the dual modes of action is to inhibit the folate pathway; the other is to permeabilize the membrane. Several sulfhydryl agents and other compounds are known to disrupt folate synthesis. Permeabilization specifically of bacterial membranes, when observed, has been less defined. Here, we showed that SCH-79797 and IRS-16 compounds permeabilize the membrane by directly activating the bacterial channel MscL. While the data are consistent with a previous report and confirm the specificity of specific compound moieties, e.g., cumene, in permeabilizing the membrane [4], our results extend the observations by demonstrating that it is the activation of MscL that is the molecular mechanism of action underlying this permeabilization. 

SCH-79797 is not the first compound found to activate the MscL channel. We previously used a high-throughput screen (HTS) to identify compounds that activated MscL [23]. This HTS was performed in vivo, using bacterial growth as the output, thus overcoming the problem of most in vitro screens that primarily find compounds that are not outer-membrane permeable, and thus ineffective for Gram-negative bacteria. Indeed, few novel antimicrobials against Gram-negative bacteria have been developed in the last decades [30]. Because MscL is in the inner membrane, permeation across the outer membrane was a requirement for seeing a compound as a positive “hit” in the MscL HTS screen. Similar to SCH-79797 and IRS-16, it is as yet unclear the pathway most of these compounds take across the outer membrane to reach the periplasm (see [31,32,33,34] for reviews). Regardless, in the original HTS, MscL expression was found to increase the potency of four previously well-characterized antibiotics [23]. Further study of one of these, dihydrostreptomycin, showed that it directly bound to, activated, and passed through the MscL channel [25]. In addition, it has recently been reported that activation of MscL is the mechanism by which the natural compound curcumin permeabilizes bacterial membranes [24]. Novel compounds from the HTS have been shown to be agonists of MscL gating, with the ability to decrease bacterial growth and increase the potency of known antibiotics by allowing the antibiotics into the cell [12,13,14] (see [35] for a recent review). However, in some of these previous examples, the efficacy and potency appeared to be somewhat limited. Here, we demonstrated that MscL activation plays an integral role in the potent and efficacious antibacterial activity of SCH-79797 and IRS-16. This finding, when combined with the discoveries that other drugs and compounds also activate MscL, strongly suggests that MscL activation should be considered as a possible and testable mechanism underlying the mode of action for compounds and drugs that have been shown to specifically permeabilize bacterial membranes by unknown mechanisms.

Dihydrostreptomycin was found to bind deep within the MscL pore, toward the cytoplasmic side. The *H. influenzae* mutational experiments, shown here for SCH-79797 in Figure 5A, are analogous to the findings of dihydrostreptomycin [25], even though the former binds more peripherally from the pore. Significantly, SCH-79797 and IRS-16 binding appears to include cytoplasmic–membrane interface regions, which is shared by other compounds that specifically activate MscL [12,13,14]. Furthermore, by using an in silico screen targeting this particular protein microenvironment, a new family of MscL-specific-activating compounds was recently discovered [36], confirming that it is indeed what has been coined a druggable protein microenvironment [37]. This region includes the S1 domain or “slide helix”, which is an amphipathic helix running along the cytoplasmic membrane, and the cytoplasmic portion of TM2, which have been previously well studied by multiple approaches [26,27,38,39,40,41] (see the review [6] for a more complete discussion). The binding sites of MscL agonists thus far are not only consistently seen toward the cytoplasmic–membrane interface but also consistently observed at the interface of assembled subunits. Such binding at subunit interfaces was first described for the nicotinic acetylcholine receptor in 1989 [42] and has since been observed for many ligands of several channels [43]. 

Although the binding regions of MscL agonist compounds are similar, the compounds themselves often have differences in their scaffolds and the understanding of how each function is important for future design. In some instances (e.g., dihydrostreptomycin, SCH-79797, and perhaps curcumin), the agonist is on the same scaffold as a moiety that works by a second mechanism (e.g., translation inhibition, folate inhibition, and the RecA pathway, respectively) while others appear to be stand-alone MscL agonists [12,13,14,36]. Martin et al. [5] found that the addition of the antibiotics Nisin [44,45] and Polimixin B [46,47], which permeabilize bacterial membranes, in combination with folate inhibitors did not lead to additive effects, and thus speculated that the two moieties leading to the two modes of action had to be on the same scaffold. This contrasts with stand-alone MscL agonists: these often do not have obviously common scaffolds; yet, all those studied thus far have been shown to improve the potencies of other antibiotics [12,13,14,36]. An obvious cause of this discrepancy is that Nisin [44,45] and Polimixin B [46,47] permeabilize membranes by other mechanisms, each with their own limitations, and do not function as MscL agonists. In sum, the data support that either engineering an MscL agonist moiety or adding an MscL agonist as an adjuvant will vastly improve an antibiotic’s potency, specificity, and ability to evade resistance. 

Although we are still at the infancy of being able to predict adjuvant efficiency or engineer motifs onto an already studied antibiotic, a better understanding of the mechanisms of different MscL agonists, and potentially how to improve efficacy and potency, will be an exciting direction for future study. MscL is an ideal antibiotic target in this respect because it is one of the best-studied MS channels and a combination of molecular dynamics and physiological experiments have already been proved to be effective tools for the prediction of agonists [36]. One day, MscL agonists may be used as adjuvants for antibiotics, or antibiotics may be modified to include MscL agonist properties by the simple addition of specific moieties. Such measures could revolutionize the field of antibiotics by reducing toxicity and acquired resistance.

## 4. Materials and Methods

### 4.1. Strains and Cell Growth

The bacterial *E. coli* Frag 1-derived strain MJF455 (ΔmscL::Cam, ΔMscS) [9] was used for all in vivo assays, and MJF612 [48] was used for the electrophysiology. The pB10d expression vector was used alone or with constructs inserted for expression. Note that this is a mid-level expression vector and in vivo expresses at only a few times more than endogenous levels [20]. For the in vivo experiments, cultures were initially inoculated from a single colony and were grown in citrate-phosphate-defined media (CphM) pH 7.0, consisting of the following per liter: 8.57 g of Na_2_HPO_4_, 0.87 g of K_2_HPO_4_, 1.34 g of citric acid, 1.0 g NH_4_SO_4_, 0.001 g of thiamine, 0.1 g of MgSO_4_∙7H2O, 0.002 g of (NH_4_)_2_SO_4_∙FeSO_4_∙H_2_O, supplemented with 0.4 mM MgSO_4_, 3 μM thiamine, 6 μM iron, 0.04% glucose, ampicillin 100 μg/mL, and incubated in a 37 °C shaker, rotated at 250 cycles per minute. 

### 4.2. In Vivo Assays

#### 4.2.1. Growth Experiments

Growth inhibition was measured as previously described [23,25]. Briefly, overnight cultures of MJF455 strain-carrying constructs were diluted 1:50 in CphM and grown until an OD_600_ of 0.2 was reached. Expression was induced by the addition of 1 mM isopropyl-b-D-thiogalactopyranoside (IPTG) for 30 min, 1 mM stocks of compound SCH-79797, IRS-10, IRS-16, or 10 mM stock of cumene were solubilized in sterile dimethyl sulfoxide (DMSO) (Sigma,-Aldrich, St. Louis, MO, USA), and diluted to two times their final concentration in pre-warmed CphM with a final DMSO concentration appropriate for their solubility of 0.9% for SCH-79797, IRS-10, and IRS-16 or 2% for cumene. We found that DMSO up to 2% does not change the growth rates for the strains used, and controls not containing drug still contained the appropriate concentration of DMSO. In total, 100 µL of the appropriate diluted compound was added to wells of a pre-warmed, sterile 96-well flat-bottom plate (Greiner bio-one, Monroe, NC, USA). Cultures were then diluted 1:200 in pre-warmed CphM, 100 µg/mL ampicillin, 2 mM IPTG, and 100 µL of culture mixture was added to the 96-well plates for a total of 200 µL, sealed with a sterile breathable film (Axygen, Union City, CA, USA), wrapped in aluminum foil and placed in a 37 °C shaker, rotated at 110 cycles per minute for 16–17 h, and OD_620_ was then taken with a Multiskan Ascent 354 (Thermo Fisher Scientific, Waltham, MA, USA) plate reader.

#### 4.2.2. Viability Experiments

Cultures were grown as above for the overnight growth experiments, and then were used for all viability experiments performed as previously described [26]. Briefly, cultures were diluted 1:20 into pre-warmed CphM, serially diluted from 10^3^ to 10^6^ in a 96-well plate, and liquid drops of 5 µL were placed on pre-warmed LB ampicillin plates and incubated overnight at 37 °C. The next morning, the colony-forming units were calculated to determine the cell viability [26].

### 4.3. Electrophysiology

*E. coli* giant cells were generated and used for patch-clamp experiments as described previously [49]. Excised, inside-out patches were examined at room temperature under symmetrical conditions using a buffer containing 200 mM KCl, 90 mM MgCl_2_, 10 mM CaCl_2_, and 5 mM HEPES pH 6 (Sigma, St. Louis, MO, USA). To study the effects of the compounds SCH-79797 and IRS-16, we used the back-filled pipette configuration, in which the pipette tip was filled with patch buffer and the back of the pipette with a solution with the desired concentration of the drug. In this configuration, which allows the drugs to be presented to the patch from the periplasmic side, patches were exercised by applying the same negative pressure as soon as the patch was formed (control) and after 30 min, allowing time for the compound to reach the tip of the pipette where the membrane patch was located. Recordings were performed at −20 mV (positive pipette). Data were acquired at a sampling rate of 20 kHz with a 5-kHz filter using an AxoPatch 200B amplifier in conjunction with Axoscope software (Axon Instruments, Union City, CA, USA). A piezoelectric pressure transducer (World Precision Instruments, Sarasota, FL, USA) was used to monitor the pressure throughout the experiments. Data were analyzed using Clampfit10 from pClamp10 software (Molecular Devices, San Jose, CA, USA).

### 4.4. Molecular Modeling and Computational Analyses

#### 4.4.1. Molecular Docking and MD Simulations

Molecular docking was performed using the Glide module of the Schrodinger’s software (Schrödinger, New York, NY, USA) [50]. Two to three top docking poses that had the distinct binding modes were selected for the subsequent molecular dynamics (MD) simulation and free energy analysis. The MD systems consisted of a copy of E. coli MscL, a ligand, 230 POPC lipids, ~0.15 M KCl, and 32,312 water molecules. The whole MD systems were described by a set of AMBER force fields, with FF14SB [51] for protein, GAFF [52] for ligands, and LIPID14 [53] for lipids, respectively. An MD system was relaxed by a series of minimization steps and constrained MD simulation steps as detailed in our previous publications [12,13,14]. Then, the system was heated up gradually in 5 100-picosecond MD simulations, with the desirable temperature being set to 50, 100, 150, 200, and 250 K, sequentially. Then, the system was equilibrated at 298 K for 15 ns prior to the sampling phase, which was conducted at 298 K, 1 bar for at least 150 ns. Integration on the equations of motion was performed at a time step of 1 fs for the relaxation phase and 2 fs for the heating, equilibrium, and sampling phases. MD snapshots were collected every 50 ps for post-analysis. All the MD simulations and the followed free energy analysis were performed using the AMBER18 software package [54].

To study the dynamics of the MscL protein and the ligands, root-mean-square-deviations of the mainchain atoms (for protein) and heavy atoms (for ligand) of the sampled MD snapshots were calculated and plotted. We also calculated the RMSD of the ligand without performing least-square fitting (the red lines in Figure S1B,D) to describe not only the conformational change in the ligand but also its translational and rotational movement inside the binding pocket. Representative MD conformations were selected as the one that had the smallest RMSD to the average structure of the MD snapshot ensemble. 

#### 4.4.2. Free Energy Analysis

For the collected MD snapshots (3000 to 4000) for the MD system, MM-GBSA decomposition was performed to identify hotspot residues that had ligand–residue interaction free energy, $∆G_{MM-GBSA}^{lig-res}$, lower than −0.1 kcal/mol. The key parameters for the MM-GBSA free energy decomposition included the internal dielectric constant, $\varepsilon_{int}=1$ and the external dielectric constant, $\varepsilon_{ext}=2$ to describe the hydrophobic part of the POPC lipids. MM-PBSA-WSAS binding free energy calculations were conducted using 10% of the snapshots evenly selected from the MD snapshot ensemble. Unlike MM-GBSA free energy decomposition analysis, two external dielectric constants, $\varepsilon_{ext}^{LIP}=2$ and $\varepsilon_{ext}^{WAT}=80$, were applied to more precisely describe the lipid bilayer and non-membrane environments, respectively. Due to the dynamic nature of the membrane, the actual thickness of the lipid and membrane center varied from one snapshot to another. Thus, two geometrical parameters, mthick and mctrdz, were calculated for individual snapshots. mthick was defined as the z-axial distance between the coordinate centers of the phosphorous atoms in the upper and lower layers of the membrane while mctrdz was defined as the z-axial distance between the coordinate center of all phosphorous atoms and the coordinate center of the MscL/ligand complex. For the ligand itself, the polar part of the solvation free energy was calculated without the implicit membrane option, i.e., only one external dielectric constant, $\varepsilon_{ext}^{WAT}=80$, was applied. The nonpolar part of the solvation free energy was estimated by the solvent-accessible surface area (SAS) using the following equation: $∆G_{nonpolar}=0.0054\times SAS+0.92$, where 0.92 is a constant. The entropic term was calculated using the WSAS method described previously [55]. 

## 5. Conclusions

This is the first instance where antimicrobial compounds that have high potency, efficacy, and a dramatic avoidance of resistance because of their dual mechanisms have been shown to target and activate a bacterial mechanosensitive channel as one of its modes of action. This finding has increased significance given that the compounds are effective against Gram-negative organisms for which new antibiotic candidates have been especially difficult to obtain. Additionally, because MscL has already been shown to be a viable drug target, engineering an MscL agonist moiety onto antimicrobials or adding an MscL agonist as an adjuvant could vastly improve an antibiotic’s potency, specificity, and ability to evade resistance.

## Data Availability

Not applicable.

## Supplementary Materials

The following supporting information can be downloaded at: https://www.mdpi.com/article/10.3390/antibiotics11070970/s1, Figure S1: Growth of *E. coli* MJF 455 cells carrying either an empty vector or expression constructs of MscL or MscS channels; Figure S2: The time courses of the root-mean-square deviations (RMSDs); Figure S3: Data indicating a binding pocket for IRS-16 to MscL; Table S1: MM-PBSA-WSAS binding free energy of the identified inhibitors of the *E. coli* MscL channel; Table S2: MM-GBSA ligand–residue interaction free energies.
