# Supplementary material for: Activation of a Bacterial Mechanosensitive Channel, MscL, Underlies the Membrane Permeabilization of Dual-Targeting Antibacterial Compounds
Source: Antibiotics (Basel). 2022 Jul 19;11(7):970. doi: 10.3390/antibiotics11070970 (PMC9312261; doi:10.3390/antibiotics11070970)
Supplement: Supplementary file 1 [file antibiotics-11-00970-s001.zip › antibiotics-1813496-supplementary.pdf]

Activating a Bacterial Mechanosensitive channel, MscL, Underlies the Membrane Permeabilization of Dual-Targeting Antibacterial Compounds

Robin Wray, Junmei Wang, Paul Blount and Irene Iscla

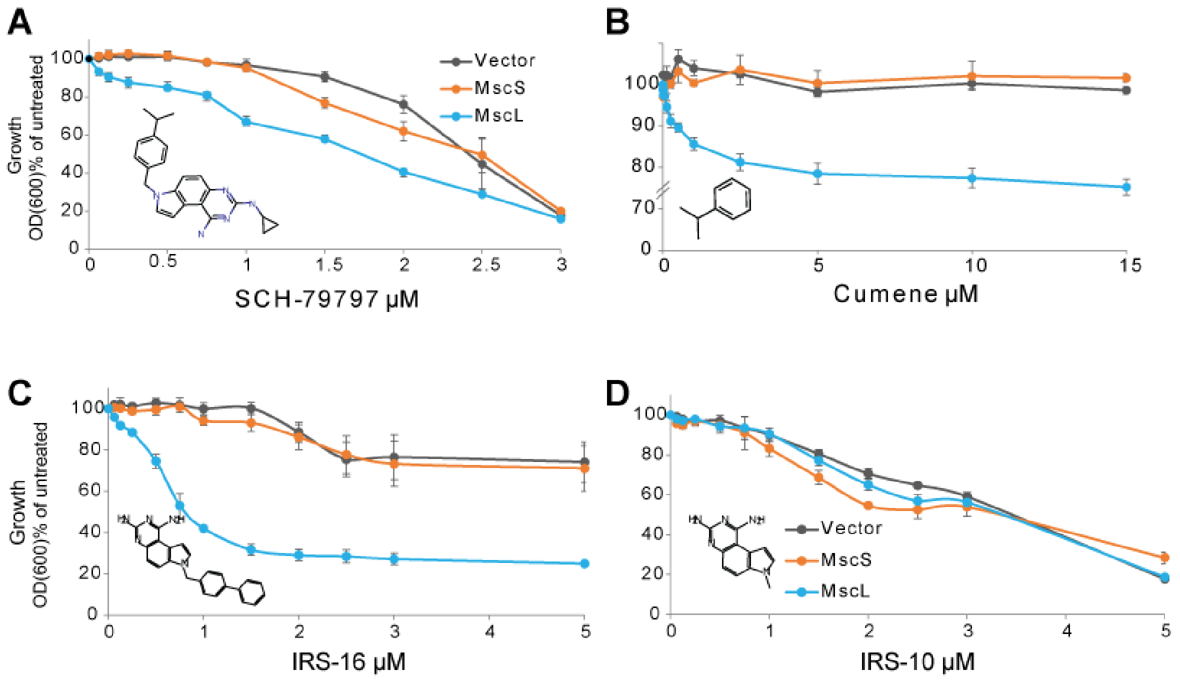

Figure S1. Growth of E. coli MJF 455 cells carrying either an empty vector or expression constructs of MscL or MscS channels. All data is represented as a percentage of the untreated sample. Cultures were treated with increasing concentrations of A. SCH-79797, B. cumene, C. IRS-16 and D. IRS-10. Structures of the compounds are depicted as inserts.

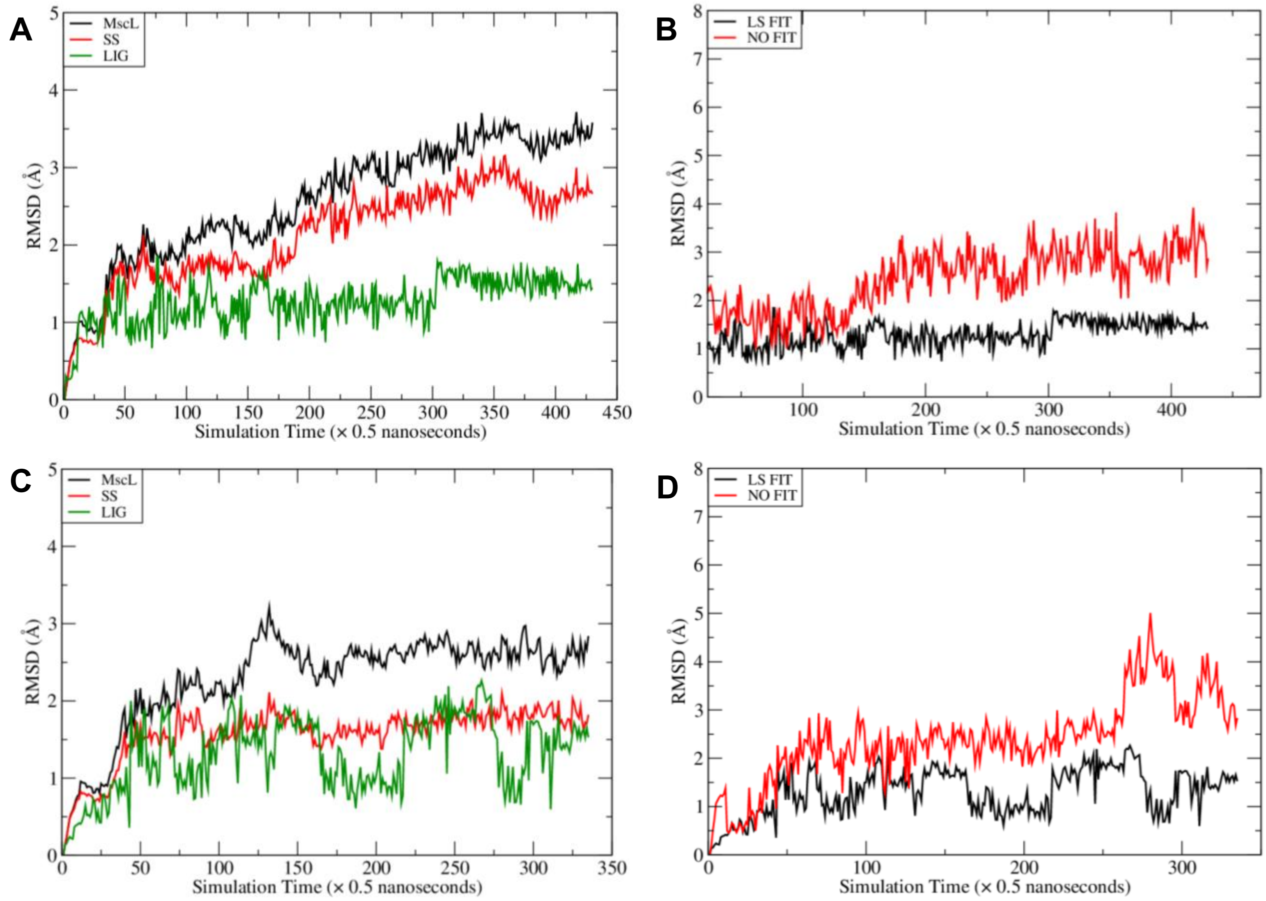

Figure S2. The time courses of Root-mean-square deviations (RMSD). A and B for SCH-79797, and C and D for IRS-16. B and D show the RMSDs of ligands with and without least-square fitting.

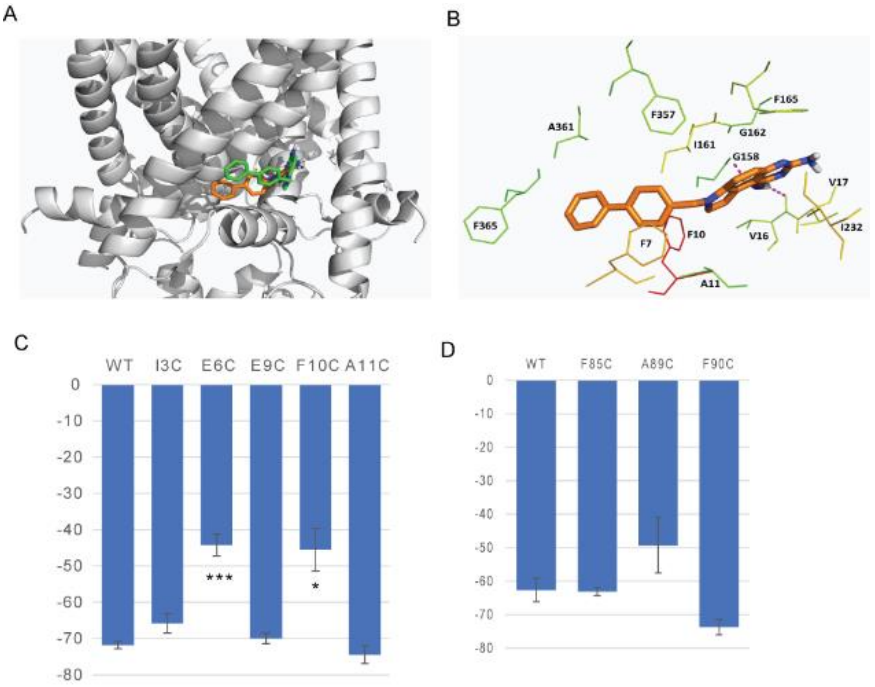

Figure S3. Data indicating a binding pocket for IRS16 to MscL. (A) Docking (greenish sticks) and representative MD (brownish sticks) binding poses for IRS16 and in vivo experiments supporting the binding site. (B) The key residues ($\Delta G_{MM-GBSA}^{lig-res} < -0.5$ kcal/mol). The more reddish a residue is, the stronger the ligand-residue interactions, the more greenish a residue is, the weaker the ligand-residue interactions. (C) Growth inhibition of MJF455 cultures expressing Eco MscL WT and N-terminal mutants, after treatment with 1μM IRS-16, expressed as a percentage of the untreated cultures. (D) C-terminal mutants. T test non paired n≥3 *p≤0.05, *** p≤0.0005.

Table S1. MM-PBSA-WSAS Binding Free Energy of the Identified Inhibitors of E. coli MscL Channel. All Energy Terms Are in kcal/mol.

**Compound**	$\Delta \boldsymbol{E}_{VDW}$	$\Delta \boldsymbol{E}_{eel}$	$\Delta \boldsymbol{G}_{sol}^{polar}$	$\Delta \boldsymbol{G}_{sol}^{nonpolar}$	**TΔS**	$\Delta \boldsymbol{G}_{MMPBSA}$
SCH-79797	-43.05 ± 1.15	-1.15 ± 0.16	9.16 ± 0.02	-4.34 ± 0.00	-21.78 ± 0.08	-23.57 ± 0.11
IRS-16	-39.59 ± 1.49	-6.9 3± 0.46	12.55 ± 0.07	-3.76 ± 0.01	-20.61 ± 0.09	-26.82 ± 0.55

Table S2. MM-GBSA Ligand-Residue Interaction Energies. Each residue is assigned a docking score value with more negative values indicating a stronger role in binding SCH-79797. All Energy Terms Are in kcal/mol. All interaction energies better than -2.0 kcal/mol were colored in red and those between -2.0 and -1.0 kcal/mol were colored in blue.

Res Type	Res ID	Res ID*	$\Delta G_{Ligand-Residue}$ (kcal/mol)	
			SCH-79797	IRS-16
ILE	3	3	-0.91	-0.27
GLU	6	6	**-1.22**	-0.47
PHE	7	7	**-2.48**	**-1.90**
GLU	9	9	-0.16	-0.21
PHE	10	10	**-1.95**	**-3.10**
ALA	11	11	-	-0.68
ARG	13	13	-	-0.29
GLY	14	14	-	-0.21
VAL	16	16	-	**-1.03**
VAL	17	17	-0.10	**-1.37**
ALA	20	20	-	-0.39
VAL	21	21	-	-0.12
ILE	24	24	-	-0.11
ASP	154	18	-0.93	-
VAL	157	21	-0.45	-
GLY	158	22	-0.28	-0.54
VAL	159	23	-	-0.18
ILE	161	25	**-2.19**	**-1.55**
GLY	162	26	-0.32	-0.82
PHE	165	29	**-1.00**	**-1.15**
ILE	228	92	-0.31	-0.34
ALA	231	95	-	-0.13
ILE	232	96	-0.54	**-1.85**
LYS	233	97	-	-0.20
PHE	357	85	**-1.18**	-0.83
LEU	358	86	-0.23	-0.40
ALA	361	89	**-1.01**	-0.82
PHE	362	90	-0.23	-0.42
ILE	364	92	-0.76	-0.20
PHE	365	93	**-1.38**	-0.57
ILE	368	96	-0.60	-
LYS	369	97	**-1.49**	-

*The corresponding residue ID in single chain of the E. coli MscL.
